# The impact of the COVID-19 pandemic on life expectancy by the level of area deprivation in South Korea

**DOI:** 10.3389/fpubh.2023.1215914

**Published:** 2023-08-01

**Authors:** Jihyung Hong, Sunghyun Yi, Taeho Yoon

**Affiliations:** ^1^Department of Healthcare Management, Gachon University, Seongnam, Republic of Korea; ^2^Department of Health Policy and Management, General Graduate School of Gachon University, Seongnam, Republic of Korea; ^3^Department of Preventive and Occupational & Environmental Medicine, College of Medicine, Pusan National University, Yangsan, Republic of Korea

**Keywords:** COVID-19, inequality, life expectancy, regional disparity, deprivation

## Abstract

**Objective:**

Comparative evidence suggests that the impact of COVID-19 on life expectancy has been relatively milder in South Korea. This study aims to examine whether the pandemic has universal or unequal impacts on life expectancy across 250 districts with varying levels of deprivation.

**Methods:**

Using mortality data from 2012 to 2021 obtained from the Microdata Integrated Service of Statistics Korea, we calculated life expectancy at birth and age 65 for both sexes, by deprivation quintiles, before and during the pandemic. We summarized life expectancy gaps using the slope of the inequality index (SII) and further decomposed the gaps by the contribution of age and cause of death using Arriaga’s method.

**Results:**

Both men and women experienced consistent improvements in life expectancy from 2012 to 2019, but the trend was disrupted during 2020 and 2021, primarily driven by older people. While men in more deprived areas were initially hit harder by the pandemic, the life expectancy gap across deprivation quintiles remained relatively constant and persistent across the study period [SII: -2.48 (CI: −2.70 from −2.27) for 2019 and − 2.84 (CI: −3.06 from −2.63) for 2020]. Middle-aged men from the most deprived areas were the most significant contributors to the life expectancy gap, with liver disease, liver cancer, transport accidents, and intentional injuries being the leading causes, both in the pre and during the pandemic. While these contributors remained largely similar before and during the pandemic, the contribution of transport accidents and liver cancer to the male life expectancy gap slightly decreased during the pandemic, while that of ischemic heart disease and pneumonia slightly increased. A similar increase was also observed for the female life expectancy gap.

**Conclusion:**

This study found no clear evidence of an increased life expectancy gap during the pandemic in South Korea, unlike in other countries, although access to emergency healthcare services may have been slightly more disturbed in deprived areas. This achievement can provide lessons for other countries. However, the persistent regional gaps in life expectancy observed over the past decade indicate the need for more targeted public health policies to address this issue.

## 1. Introduction

South Korea has achieved a remarkable improvement in life expectancy from 62.2 years in 1970 to 83.2 years in 2019, placing it among the countries with the highest life expectancy globally (1). Additionally, South Korea is also one of the few nations, including Denmark, Iceland, and New Zealand, where life expectancy remained relatively unchanged during the COVID-19 pandemic (2, 3) (see Supplementary Figure A1 for the overall trend of life expectancy and mortality rate, Supplementary material). Despite being one of the first countries affected by the pandemic, South Korea managed to effectively control multiple waves of COVID-19 through a proactive testing-contact tracing-treatment (3 T) strategy. Compared to peer high-income countries, South Korea had significantly lower cumulative incidence rates of confirmed cases, with 1,172 per million people in 2020 and 12,258 in 2021 (as of Dec 31), and comparatively low mortality rates throughout the pandemic, with 17.4 per million people in 2020 and 90.0 in 2021 (4).

However, the impact of the pandemic goes beyond the COVID-19 incidence and mortality rates (5). Healthcare resources were inevitably redirected toward combating COVID-19, possibly resulting in some disruption to existing healthcare services, particularly in emergency services and severe illness treatments. Such disruption is likely more pronounced in areas and groups where healthcare provision is already limited or vulnerable. Furthermore, strict social distancing measures and heightened border controls, both locally and internationally, have significantly impacted many industries and small businesses, particularly those that were already vulnerable (5). For instance, non-regular workers experienced higher job losses and pay cuts than their counterparts during the pandemic (5). Such unequal impacts of COVID-19 can subsequently widen socioeconomic gradients in health. For example, Oh et al. found a positive correlation between lower socioeconomic status and a higher risk of contracting COVID-19 in South Korea (6). Similarly, Yun et al. reported stronger associations between monthly household income and perceived health during the pandemic than in the pre-pandemic era, based on survey data from 2018 and 2021 (7).

Therefore, the impact of COVID-19 on life expectancy may also vary among different population segments, which could differ from an overall country trend. International evidence suggests that the incidence and mortality rates due to COVID-19 are disproportionately higher among marginalized communities and individuals (8, 9). Specifically, a recent review by McGowan and Bambra presented mounting evidence (*n* = 86) suggesting that areas of socioeconomic disadvantage experienced higher COVID-19 mortality rates compared to affluent areas (10). For example, mortality rates of COVID-19 in the United States were more than twice as high in low-income counties than in the wealthiest ones (11). Moreover, the excess mortality rate from COVID-19 was higher in counties with higher poverty rates and lower levels of education, and it was the highest in counties with the highest percentage of Black residents (3). In addition, a Chilean study reported a greater decline in life expectancy in poorer urban areas, compared to the previous 5 years (12).

Despite South Korea’s relatively successful response to the COVID-19 pandemic with a comparably low rate of COVID-19 incidence and mortality, it is important to investigate whether mortality impacts have been consistent across all regions, including those with limited access to healthcare services and social safety nets. This study, therefore, aims to explore the impact of COVID-19 on life expectancy among different population segments in South Korea, encompassing both direct mortality effects attributed to the virus and indirect mortality effects resulting from public health interventions and broader societal responses. Specifically, it analyzes and compares life expectancy at birth and age 65, by sex, before and during the COVID-19 pandemic in areas (*si-gun-gu*) divided into quintiles of the area deprivation index. The study also attempts to quantify how deaths from specific causes and at different age groups contribute to disparities in life expectancy between the most deprived and affluent areas and assess whether the contribution of causes of death and age groups changed during the pandemic compared to the previous 5 years (2015–2019). By examining these factors, the study can determine if the impact of COVID-19 on life expectancy is consistent across various levels of deprivation and inform public health policy.

## 2. Materials and methods

### 2.1. Data

Mortality data from 2012 to 2021 were extracted from the Microdata Integrated Service (MDIS) of Statistics Korea (13). The data includes information on de-identified deceased individuals, such as their sex, age, place of death, and cause of death. These data were supplemented with official age-specific population counts (14). The cause of death was recorded using the Korean Standard Classification of Diseases based on the International Classification of Diseases 10th version (ICD-10) (see Table A1, Supplementary material) (15). Deaths and leading specific causes of death with a mortality rate of at least 10 per 100,000 in either 2012 or 2021 (16) were stratified by sex, age group (excluding ages 0, 1–4, and over 90), and area units (*si-gung-gu*).

### 2.2. Deprivation index

We utilized the publicly available deprivation index from the Busan Public Health Policy Institute (17), which was constructed using various indicators of individual and community welfare and well-being at the district (*si-gun-gu*) level (*n* = 250) from the 2020 population census data. Based on the 2020 deprivation index, all districts were classified into quintiles of area deprivation (see Supplementary Figure A2), and we used the same quintile grouping throughout the study period. This allowed for consistent tracking of changes in mortality rates and life expectancy over time in the same district groups. In addition, using year-specific quintiles was not feasible because the deprivation index is updated every 5 years based on new population census data. It is worth noting that the 2015 and 2020 deprivation indices showed a very high correlation (*r* = 0.98), indicating that deprivation and affluence persist over time in South Korea.

### 2.3. Statistical analysis

We created abridged life tables with 5-year age intervals (except 0 years old and 1 ~ 4 years old) up to 90+ years old for each sex at the district level (*si-gun-gu*; see Supplementary Figure A3) and quintiles of area deprivation index for the period of 2012–2021, using Chiang’s methods (18, 19). To assess the impact of the COVID-19 pandemic on mortality and life expectancy in different age groups, we also calculated the probability of not surviving up to 65 for a newborn and up to 85 for a 65-year-old, using the abridged life tables (i.e., the product of age-specific (conditional) survival probabilities or the number alive at the beginning of the age interval) (12). The slope index of inequality (SII), a widely used measure of health inequality, was used to summarize the difference in life expectancy between the most and least deprived areas. It was calculated using linear regression, allowing for differences in population size between deprivation quintiles. The index can be interpreted as the absolute difference in life expectancy between the most deprived and affluent areas.

In addition, we employed Arriaga’s method to decompose the life expectancy gap by age and cause of death between the most deprived and affluent quintiles (20–23). As shown in Table A1 (Supplementary material), the cause of death includes 22 categories, including the leading causes and the rest categories (e.g., ‘all other cancers’). The method first assessed how much the mortality differences of each age group between the two populations affected the years of life lived at that age (direct effects) and subsequent age (indirect and interaction effects) to determine the age-specific contribution to the life expectancy gap as shown in Equation 1:


(1) $$\begin{array}{c} \mathrm{nCx}=\left[\frac{l_{x}^{deprived}}{l_{0}}\times \left(\frac{nL_{x}^{affluent}}{l_{x}^{affluent}}-\frac{nL_{x}^{deprived}}{l_{x}^{deprived}}\right)\right]\\ +\left[\frac{T_{x+n}^{affluent}}{l_{0}}\times \left(\frac{l_{x}^{deprived}}{l_{x}^{affluent}}-\frac{l_{x+n}^{deprived}}{l_{x+n}^{affluent}}\right)\right] \end{array}$$

where nCx is the total contribution (in years) in age group x to x + n, $l_{x}$ , nLx and $T_{x}$ are conventional functions of the life table calculated earlier. More specifically, $l_{x}$ is the number of people alive at age x in a hypothetical cohort, $l_{0}$ is the cohort size at the start (100,000 in a life table), nLx is the number of person-years lived between ages x and x + n, and $T_{x+n}$ is the total number of person-years lived above age x + n.

The first term of Equation 1 represents the direct effect of age, which is the number of years an age group adds to a life expectancy gap, driven by difference in mortality rates in that specific age group between the two populations. The second term of Equation 1 indicates an indirect/interaction effect, which refers to the effect placed on all later age groups–like domino effects–led by the direct effect. The sum of nCx across all age groups should be equal to the difference in life expectancy at birth between the most deprived and affluent areas.

This age-specific contribution ( nCx ) can also be decomposed by cause of death. The contribution ( $nC_{x}^{i}$ ) of cause *i* to the age-specific contribution can be estimated with the Equation 2:


(2) $$nC_{x}^{i}=\mathrm{nCx}\times \left[\frac{nR_{x}^{i,affluent}\times nm_{x}^{affluent}-nR_{x}^{i,deprived}\times nm_{x}^{deprived}}{nm_{x}^{affluent}-nm_{x}^{deprived}}\right]$$

where nRx is the proportion of deaths from cause *i* in age group x to x + n and nm_x_ is the all-cause mortality rate in that age group. The total contribution of any given cause to the life expectancy gap can be obtained by summing cause-specific contributions across all age groups.

We compared the results between the pre-COVID-19 period (2015–2019) and the COVID-19 period (2020–2021) to identify changes in the contribution of age groups and causes of death to the life expectancy gap during the pandemic.

All analyses were conducted using the R software (version 4.3.0) and can be replicated using R packages ‘PHEindicatormethods’ (life tables and related probabilities, SII) and Arriaga’s macro developed by Auger et al. (20).

This study utilized publicly available, de-identified mortality data, which met the criteria for exemption from ethics review as determined by the Institutional Review Board of Gachon University.

## 3. Results

### 3.1. Life expectancy during 2012 ~ 2021

**Figure 1:**
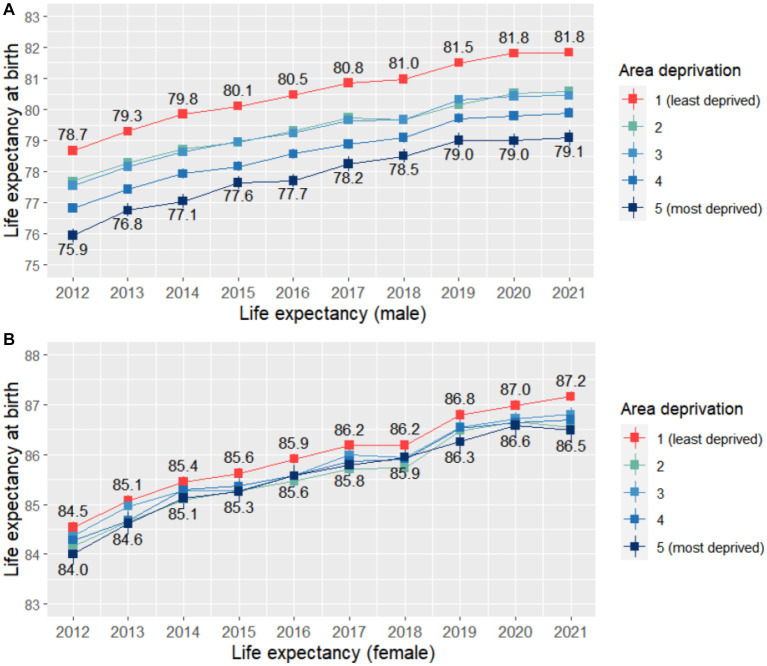
Life expectancy at birth by quintiles of deprivation index during 2012 ~ 2021 (by sex).

We first examined trends in life expectancy for men and women across different levels of area deprivation, using data from 2012 to 2021. As shown in Figure 1, both men and women, regardless of where they live, experienced steady improvements in life expectancy from 2012 to 2019. However, men from the most affluent areas consistently had higher life expectancy than those from the most deprived areas. For instance, in 2012, men from the most affluent areas had a life expectancy of 78.7 years (95% CI, 78.5 ~ 78.8), 2.8 years longer than men from the most deprived areas (LE: 75.9, 95% CI: 75.7 ~ 76.2). Similarly, men aged 65 from the most affluent areas were expected to live 0.9 years longer than their counterparts (Supplementary Figure A4). While women from the most affluent areas also had higher life expectancy, the pattern was less clear than for men, and there were no longer gaps by deprivation quintile for women aged 65.

However, the COVID-19 pandemic had a notable impact on life expectancy in 2020 ~ 2021, with the increasing trend generally no longer observed during this period. While the impact of COVID-19 on life expectancy was still broadly similar across deprivation quintiles, men from more deprived areas appeared to be hit first by the pandemic. This was somewhat reflected in the Slope Index of Inequality (SII) for life expectancy, which numerically increased slightly from 2019 (SII: -2.48, 95% CI: −2.70 ~ −2.27) to 2020 for men (SII: -2.84, 95% CI: - 3.06 ~ −2.63; Supplementary Figure A5).

**Figure 2:**
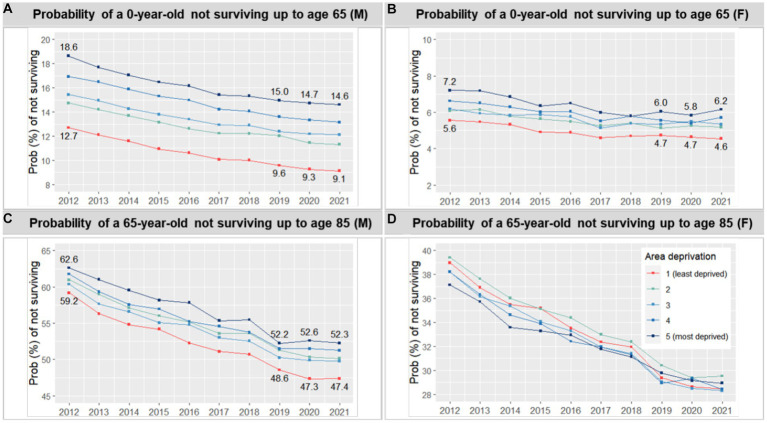
Association between life expectancy at birth and area deprivation at the district level during 2015 ~ 2019 and 2020 ~ 2021 (by sex).

We conducted district-level analyses as part of our robustness checks to further examine the relationship between area deprivation and life expectancy. The visual examination confirmed a negative correlation between deprivation and life expectancy, both before and during the pandemic, with correlation coefficients of −0.66 to −0.67 for men. However, the relation was less clear for women, with correlation coefficients of −0.11 to −0.19. Notably, male life expectancy not only showed systematic differences across deprivation quintiles but also appeared to be more homogeneous within each quintile compared to female life expectancy, as shown in the density distributions presented in Figure 2.

**Figure 3:**
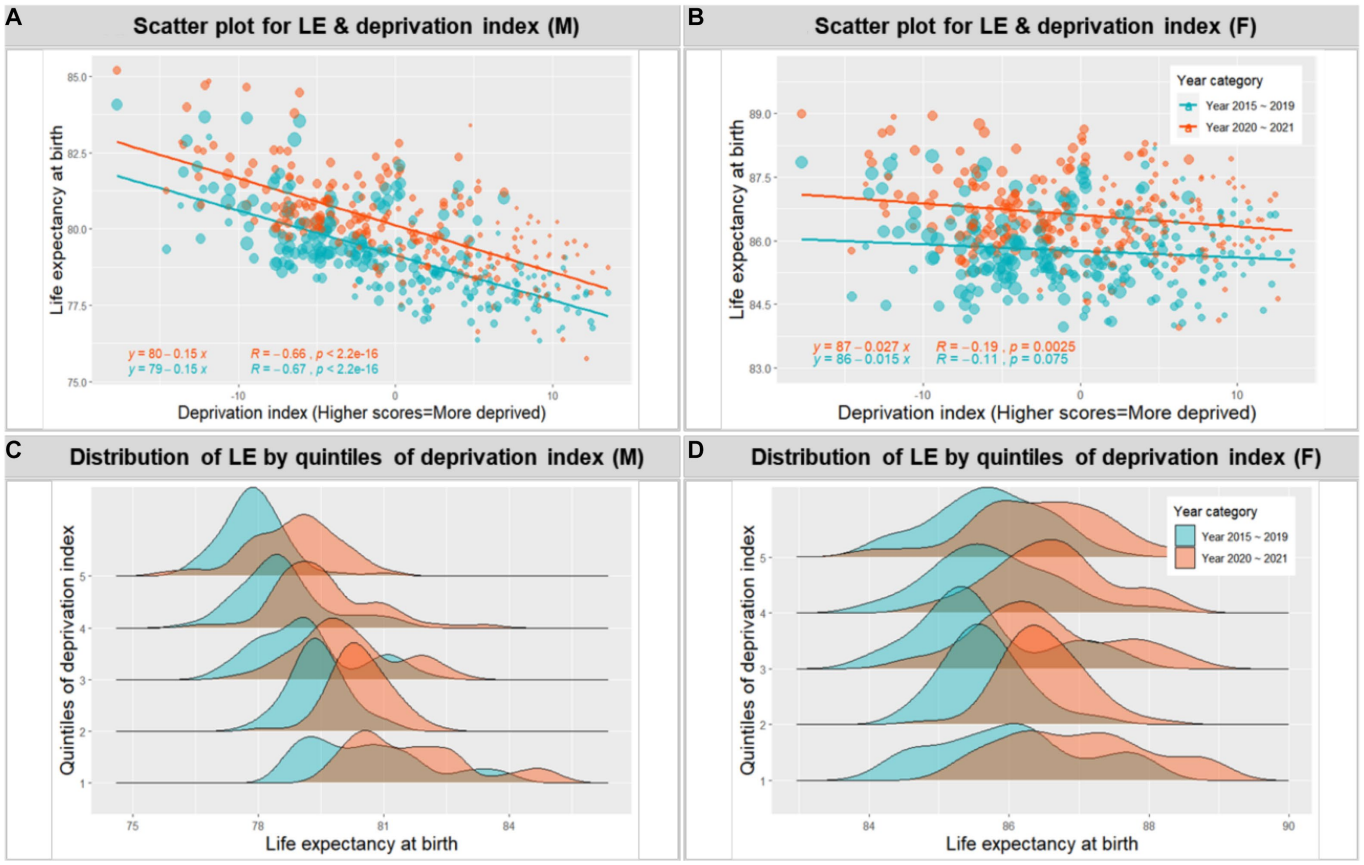
Probability (%) of not surviving up to a given age, by sex.

In addition, we estimated the probability of not surviving up to age 65 and up to age 85 for both men and women to explore the impact of the pandemic on different age groups (Figure 3). The results showed that consistent with the trend of increasing life expectancy from 2012 to 2019, the probability of not surviving up to a given age decreased during this period. However, the trend for the probability of a 65-year-old not surviving up to age 85 was curbed during the COVID-19 pandemic for both men and women. This effect was not evident for the probability of a 0-year-old not surviving up to age 65.

### 3.2. Contribution of age groups and causes of death to the life expectancy gap

**Figure 4:**
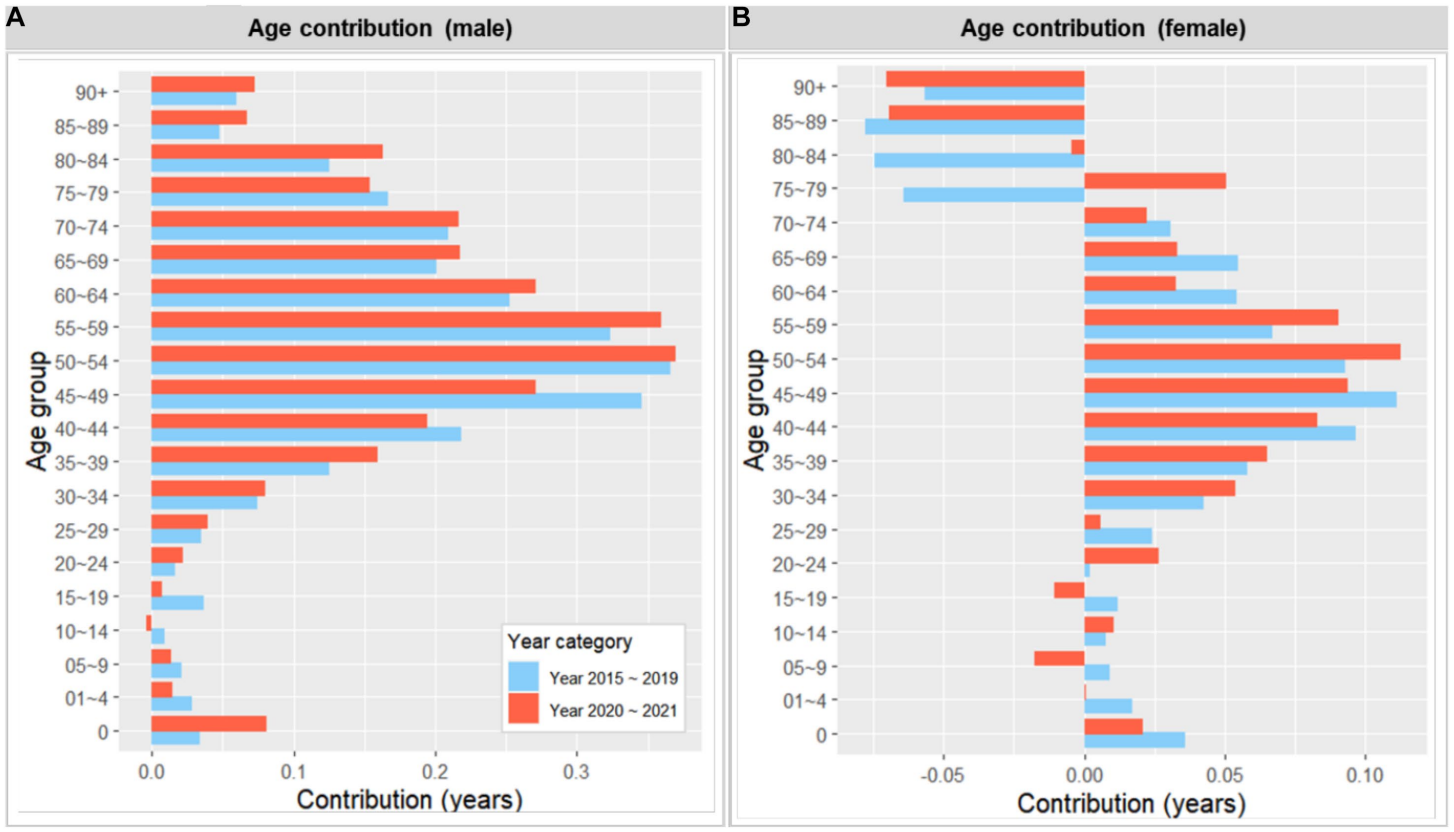
Number of years contributed by age to the life expectancy gap between the most deprived and the least deprived areas, 2015 ~ 2019 and 2020 ~ 2021 (by sex). A positive value indicates a contribution from the most deprived area (i.e., greater mortality).

**Figure 5:**
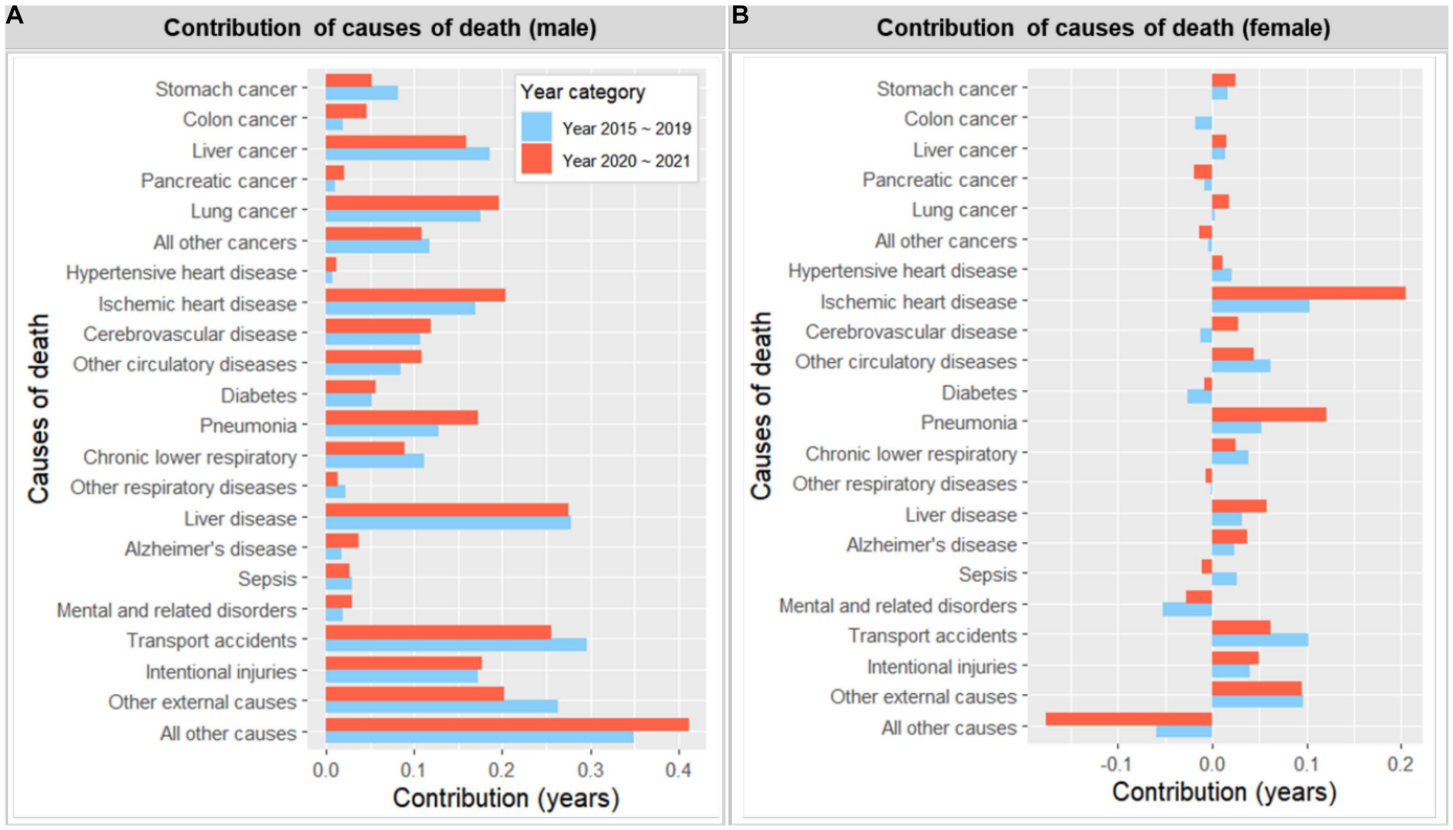
Number of years contributed by cause of deaths to the life expectancy gap between the most deprived and the least deprived areas, 2015 ~ 2019 and 2020 ~ 2021 (by sex). A positive value indicates a contribution from the most deprived area (i.e., greater mortality).

Figures 4, 5 illustrate the years that different age groups and causes of death contributed to the life expectancy gap between the most deprived and affluent areas, for both men and women, during the periods of 2015 ~ 2019 and 2020 ~ 2021. Higher mortality rates in the most deprived areas across almost all age groups, particularly those in their 40s and 50s, contributed to the male life expectancy gap, both in the pre-and during COVID-19 periods. Although the pattern of age contribution was similar across the two periods, the contribution from 0 years old slightly increased during the pandemic (from 0.034 years to 0.081), while that from 45 ~ 49 slightly decreased during the same period (from 0.346 years to 0.271). In contrast, the pattern for female life expectancy was less clear. Although higher mortality rates in the most deprived areas generally contributed to the life expectancy gap, this effect was partially offset by higher mortality rates among older women in the most affluent areas. Notably, the offset age increased from 75 ~ 79 in 2015 ~ 2019 to 85 ~ 89 in 2020 ~ 2021. In general, the contribution of men and women in their 40s to the life expectancy gap slightly decreased during the pandemic.

In relation to the contribution of causes of death, higher mortality rates in the most deprived areas, across all causes of death examined, contributed to the male life expectancy gap in both periods. The leading causes of death that contributed to the life expectancy gap during the pre-pandemic 5 years were transport accidents (0.296 years), liver disease (0.278), liver cancer (0.19), lung cancer (0.185), intentional injuries (0.173) and ischemic heart disease (0.170), and pneumonia (0.128). Although this pattern remained largely similar during 2020 ~ 2021, the contribution of transport accidents (0.256) and liver cancer (0.160) slightly decreased, while that of ischemic heart disease (0.204) and pneumonia (0.172) slightly increased during the same period. In contrast, the effect of causes of death was again not as clear for the female life expectancy gap. The contribution from the most deprived area during the pre-pandemic era included ischemic heart disease (0.103 years), transport accidents (0.101), and pneumonia (0.052), while that from the most affluent areas during this period included mental, behavioral, and neurodevelopmental disorders (−0.052), and this pattern remained largely similar during 2020 ~ 2021. However, ischemic heart disease (from 0.103 to 0.204 years) and pneumonia (from 0.052 to 0.121 years) made a small but notable increase in contribution during the latter period.

Supplementary Figures A6, A7 visually display the contribution of causes of death and age combinations to the life expectancy gap. Consistent with the above findings, middle-aged men from the most deprived areas, particularly in liver disease, followed by liver cancer, transport accidents, and intentional injuries, contributed the most to the life expectancy gap. While such a pattern was not apparent for women, older women from the most affluent areas also made a notable contribution due to other causes of death unspecified.

## 4. Discussion

This study explores the equity implications of COVID-19 on life expectancy in South Korea, specifically examining whether the overall trend of life expectancy, which has been relatively stable during the pandemic (2, 3), has masked any unequal effects of COVID-19 across different areas of deprivation. The findings indicate that, unlike in other countries (12, 24), the overall trend of life expectancy remained largely consistent across area deprivation quintiles during the pandemic, suggesting no significant worsening of regional gaps. However, the results also underscore a persistent regional gap in life expectancy, particularly for men, over the past decade in South Korea. The study further examines the contributions of age groups and causes of death to these regional gaps in life expectancy, both before (2015–2019) and during the pandemic (2020–2021). The contributions of these factors remained mostly similar between the pre-pandemic era and the pandemic period, although some discrepancies were observed. Compared to the pre-pandemic era, the contribution of transport accidents and liver cancer to the male life expectancy gap slightly decreased during the pandemic, while the contribution of ischemic heart disease and pneumonia slightly increased. The following section provides a more comprehensive discussion of the study findings from a public health perspective.

### 4.1. Life expectancy gaps and COVID-19

The COVID-19 pandemic has had a disproportionate impact on disadvantaged populations in many countries, leading to higher COVID-19 mortality and poorer health outcomes such as anxiety in these groups (8–11). While some studies have primarily focused on COVID-19 mortality rates across different population segments, others have taken a broader approach by examining all-cause mortality and life expectancy, given the pandemic’s wider public health and societal implications (2, 3). Although these studies have mostly been conducted on a global scale, recent studies in Chile (12) and England and Wales (24) have shown that COVID-19 has exacerbated existing inequalities within countries in terms of life expectancy and related overall outcomes. The Chilean study found that poorer urban areas had a ‘double burden’ in terms of life expectancy, with lower levels of life expectancy and greater declines compared to the previous 5 years (12). Similarly, Kontopantelis et al. reported that during the first 42 weeks of the pandemic in England and Wales, the most deprived areas experienced almost twice as many excess years of life lost (YLL) compared to the most affluent areas, partly due to the pandemic’s impact on younger age groups (24).

While our study also found persistent inequalities in life expectancy across different levels of area deprivation from 2012 to 2021, we did not observe clear evidence of an increased life expectancy gap during the pandemic. This could be attributed, in part, to the effective response of the Korean government to COVID-19, which drew on lessons learned from the 2015 MERS outbreak (25). Similar to the MERS outbreak, the government implemented a zero-COVID-19 policy during the first wave of the pandemic in 2020 but subsequently shifted to a sustainable suppression policy with a systematic and proactive 3 T strategy and social distancing measures, without resorting to within-border lockdowns. Prompt and transparent communication with the public may have also helped promote public awareness and cooperation in wearing masks, following guidelines, and assisting with contact tracing, either directly or partially through increased social pressures. Additionally, South Korea’s high number of hospital beds (12.7 per 1,000 population in 2020) (26), the highest among OECD countries, may have helped better manage COVID-19 cases, although the lengthy admission practice enabled by such capacity was a concern before the pandemic.

Furthermore, it is worth noting that South Korea may have more effective systems in place to mitigate the impact of external shocks on mortality and life expectancy across different areas of deprivation. In comparison to other countries, underlying health inequalities in terms of life expectancy were relatively less severe in South Korea, although a nearly three-year gap in male life expectancy persisted throughout the study period. For instance, in England, male life expectancy at birth was 73.5 years in the most deprived areas between 2018 and 2020, nearly 10 years shorter than in the least deprived areas (83.2 years) (27). However, it is important to consider that the unequal impact of COVID-19 on health outcomes in South Korea may not have yet resulted in clear disparities in life expectancy, given that existing evidence indicates an increase in socioeconomic gradients in health during the pandemic (e.g., perceived health and the risk of contracting COVID-19) (6, 7). Continued monitoring is imperative to assess the long-term health effects of COVID-19 on different population segments.

Our study also confirms that the stalled increase in life expectancy during the pandemic was primarily due to disturbed mortality among older people, a phenomenon observed globally. Despite relatively low mortality rates of COVID-19, about 95% of COVID-19 deaths in 2020 were among people aged 60 or older (28), with a significant proportion of them residing in group living facilities such as nursing homes (29). A disproportionately higher mortality rate among older people, compared to the pre-pandemic era, is not only because they faced a higher risk of mortality from COVID-19 but also possibly due to other causes of death related to disrupted healthcare services. Therefore, better-targeted policies are necessary, particularly for vulnerable groups such as those residing in group residences, as similar pandemics are likely to occur periodically in the future.

### 4.2. Decomposition of the life expectancy gap by age and leading causes of death

We also investigated how age groups and leading causes of death contributed to the difference in life expectancy between the most deprived and affluent areas during the COVID-19 pandemic and compared these findings to those from the pre-pandemic era. The findings revealed that the pattern of age groups and causes of death contributing to the gap during the pandemic was similar to that observed in the previous 5 years. The gap remained more prominent among men, primarily driven by middle-aged men and chronic conditions such as liver disease, liver cancer, lung cancer, ischemic heart disease, and external causes such as transport accidents and intentional injuries. Interestingly, compared to the pre-pandemic era, the contribution of transport accidents and liver cancer to the male life expectancy gap slightly decreased during the pandemic, while that of ischemic heart disease and pneumonia slightly increased. A similar pattern of increase was also observed for the female life expectancy gap.

Several factors may have contributed to these changes during the pandemic. Firstly, although our study did not observe an increase in life expectancy gap between deprived and affluent areas during the pandemic, access to certain health services, such as emergency care, may have been disrupted, particularly in more deprived areas where such facilities are already limited. For instance, Sung et al. reported a decrease in emergency department visits for acute myocardial infarction during the early 2020 outbreak period compared to the same period in the preceding 2 years, possibly due to misinterpretation of symptoms, fear of contracting COVID-19, and limited access to emergency healthcare services (30). Such disruptions could have been more pronounced in more deprived areas, as indicated by our findings that the contribution of emergency medical conditions - such as ischemic heart disease and possibly cardiovascular disease - to the life expectancy gap increased during the pandemic. Additionally, COVID-19 can cause lung complications like pneumonia, which can again disproportionately affect older people in more deprived areas. Meanwhile, the contribution of transport accidents and liver cancer slightly decreased during the pandemic, possibly driven by social distancing measures. The implementation of social distancing measures, coupled with societal pressures, led to an increase in remote work and a decrease in social gatherings. This led to reduced movement and alcohol consumption, as indicated by a recent survey that examined the public’s attitudes toward alcohol during the pandemic (31).

While direct comparisons between findings from different countries pose challenges, as other studies primarily focused on changes in mortality by cause of death rather than changes in the contribution of cause of death to the life expectancy gap, several studies have highlighted similar shifts in specific causes of death (32, 33). For instance, in Italy, Grande et al. observed an increase in mortality related to pneumonia and other respiratory diseases during the pandemic, potentially influenced by underreported COVID-19 cases (32). They also found a decrease in mortality related to transport accidents, as reported in Peru (33). Both studies underscored the impact of national lockdown measures on transport accidents during the pandemic.

It should also be noted that the underlying causes of inequality, although not attributed to the pandemic itself, were largely related to unhealthy behavior, such as alcohol consumption, smoking, and poor diets. It has been well documented that unhealthy behavior is more prevalent among disadvantaged populations. For instance, Chang et al. reported persistent socioeconomic inequalities in smoking prevalence between 1992 and 2016, although the prevalence itself among men decreased from 71.7% in 1992 to 39.7% in 2016 (34). Furthermore, Kim and Jung-Choi’s research found that high-risk alcohol drinking, smoking, and physical inactivity were more prevalent among people with lower incomes, and similar patterns were observed for hypertension, diabetes, and obesity (35). Although addressing the root causes of mortality inequalities is complex, targeted public health interventions can be effective in tackling unhealthy behaviors.

### 4.3. Limitations

Our study has several limitations that should be taken into account when interpreting the results.

Firstly, although life expectancy is a comprehensive measure of population health, it may not be very sensitive enough to detect subtle differences in the impact of COVID-19 on different groups, particularly when mortality rates are low. Therefore, it is important to monitor other intermediate health outcomes in conjunction with life expectancy to gain a more precise understanding of health inequalities. Furthermore, inequality in terms of life expectancy could manifest over time, and therefore continuous monitoring is required to track changes over time.

Secondly, South Korea was more severely affected by the Omicron variant during early 2022, but this period was not included in our analysis due to the unavailability of mortality data. Future research should include this period to examine the full impact of COVID-19 on life expectancy gaps when data becomes publicly available.

Thirdly, while deprivation is strongly linked to mortality, it cannot entirely account for the variation in area-level mortality rates and life expectancy. The deprivation index used in this study had a correlation of approximately 0.6 with male life expectancy, but measurement errors may also have contributed to the imperfect relationship. Although the development of the deprivation index is constrained by data availability, there is room for enhancements to more effectively identify areas facing deprivation.

Finally, in order to increase the number of cases available for analysis, districts were grouped into quintiles rather than deciles due to a low number of observed deaths, particularly in the decomposition analysis. Nonetheless, robustness checks using deprivation deciles yielded largely consistent results.

## 5. Conclusion

Despite the COVID-19 pandemic having unequal implications across different population segments, this study does not provide definitive evidence of an increased gap in life expectancy during this period in South Korea, which contrasts with findings in other countries. Although certain disruptions caused by COVID-19, such as limited access to emergency healthcare services, may have had a more pronounced impact in deprived areas, the overall findings suggest that life expectancy remained largely stable during the pandemic, and regional disparities in life expectancy did not worsen. Consequently, South Korea’s proactive approach in managing the pandemic, which involved extensive testing, contact tracing, and treatment strategies, can offer valuable insights and lessons for other nations in preparing for and responding to future pandemics. Nevertheless, the persistent regional gaps in life expectancy observed over the past decade, particularly among men, underscore the ongoing need for targeted public health policies to address this pressing issue.

## Data availability statement

Publicly available datasets were analyzed in this study. This data can be found at: https://mdis.kostat.go.kr/index.do and http://www.bhi.or.kr/bppi/view.do?no=160.

## Ethics statement

The study used publicly available, de-identified mortality data, which made it eligible for an exemption from ethics review by the Institutional Review Board of Gachon University.

## Author contributions

JH designed the study, conducted the statistical analysis, and drafted the manuscript. SY assisted with data cleaning and statistical analyses. TY contributed to the study design and provided a critical review of the manuscript. All authors contributed to the article and approved the submitted version.

## Funding

This work was supported by the Gachon University research fund of 2020 (GCU-2020- 202002900001).

## Conflict of interest

TY served as the Director-General of Public Health Policy, overseeing the coordination and implementation of COVID-19 pandemic responses in the Ministry of Health and Welfare until June 2021.

The remaining authors declare that the research was conducted in the absence of any commercial or financial relationships that could be construed as a potential conflict of interest.

## Publisher’s note

All claims expressed in this article are solely those of the authors and do not necessarily represent those of their affiliated organizations, or those of the publisher, the editors and the reviewers. Any product that may be evaluated in this article, or claim that may be made by its manufacturer, is not guaranteed or endorsed by the publisher.
