# Supplementary material for: The impact of the COVID-19 pandemic on life expectancy by the level of area deprivation in South Korea
Source: Front Public Health. 2023 Aug 1;11:1215914. doi: 10.3389/fpubh.2023.1215914 (PMC10427859; doi:10.3389/fpubh.2023.1215914)
Supplement: Supplementary file 1 [file Table_2.DOCX]

Supplementary Material

**The impact of the COVID-19 pandemic on life expectancy by the level of area deprivation in South Korea**

Jihyung Hong^*^, Seong-Hyun Yi, Taeho Yoon

*** Correspondence:** Jihyung Hong: [jihyung.hong.kr@gmail.com](mailto:jihyung.hong.kr@gmail.com)

## Figure A1. The overall trend of life expectancy at birth and age-adjusted mortality rate during 2012-2021


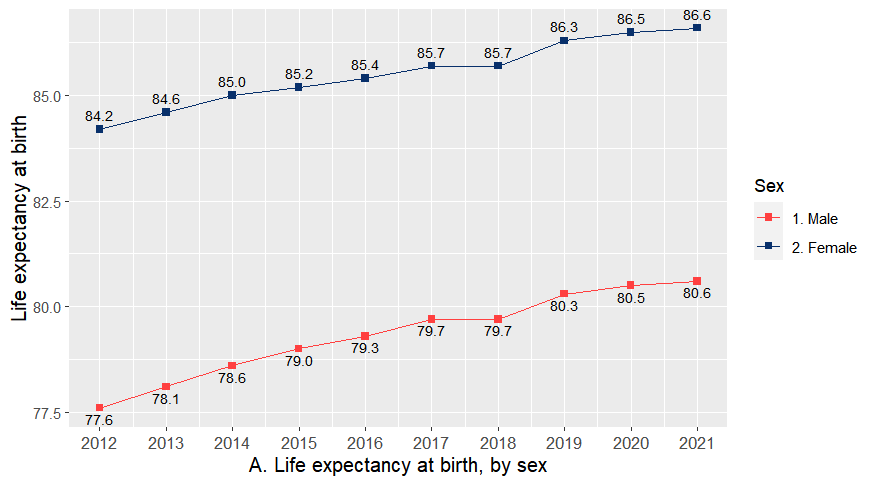


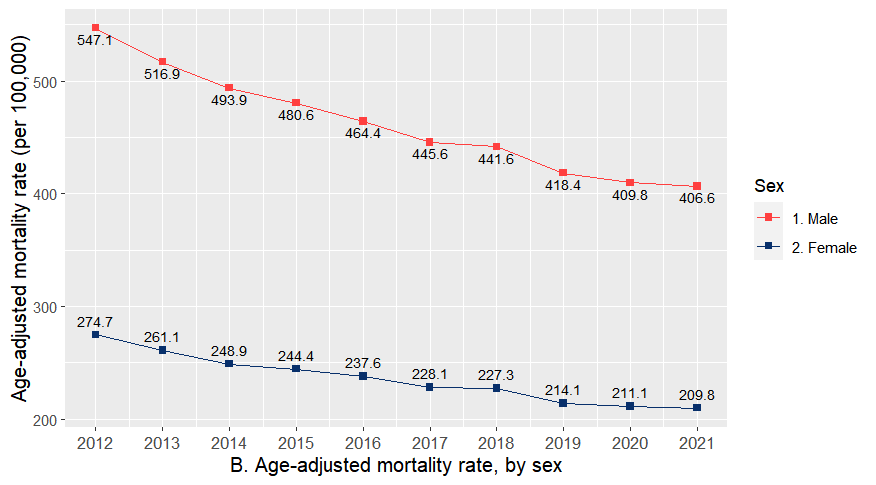


**Note**: These figures represent official life expectancy at birth and age-adjusted mortality rate reported by Statistics Korea.

- Korean Statistical Information Service. Cause of death. (2023). Available online at: <https://kosis.kr/statHtml/statHtml.do?orgId=101&tblId=DT_1B34E13&conn_path=I3> (accessed June 2, 2023).

- Statistics Korea. Life table 2021. (2023). <https://www.index.go.kr/unify/idx-info.do?idxCd=8016>) (accessed June 2, 2023).

## Table A1. International Classification of Disease (ICD-10) codes for causes of death

| **Cause of death** | **ICD-10** |
| --- | --- |
| **Cancer** | C00-C97 |
| Stomach cancer | C16 |
| Colon cancer | C18-C21 |
| Liver cancer | C22 |
| Pancreatic cancer | C25 |
| Lung cancer | C33-C34 |
| All other cancers | - |
| **Circulatory diseases** | I00-I99 |
| Hypertensive heart disease | I10-I13 |
| Ischemic heart disease | I20-I25 |
| Cerebrovascular disease | I60-I69 |
| Other circulatory diseases | - |
| Diabetes | E10-E14 |
| **Respiratory diseases** | J00-J98, U04 |
| Pneumonia | J12-J18 |
| Chronic lower respiratory diseases | J40-J47 |
| Other respiratory diseases | - |
| Liver diseases | K70-K76 |
| Alzheimer's disease | G30 |
| Sepsis | A40-A41 |
| **Mental, Behavioral and Neurodevelopmental disorders** | F01-F99 |
| **External causes** | V01-Y89, U12 |
| Transport accidents | V01-V99 |
| Intentional injuries | X60-X84 |
| Other external causes | - |
| **All other causes** | - |

## Figure A2. A geographic distribution of deprivation index in 2020

**
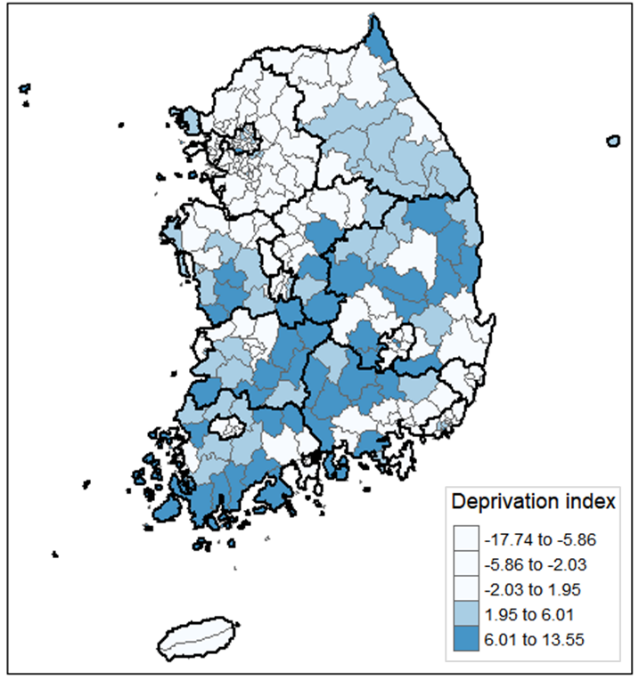
**

## Figure A3. A geographic distribution of life expectancy at birth in 2020


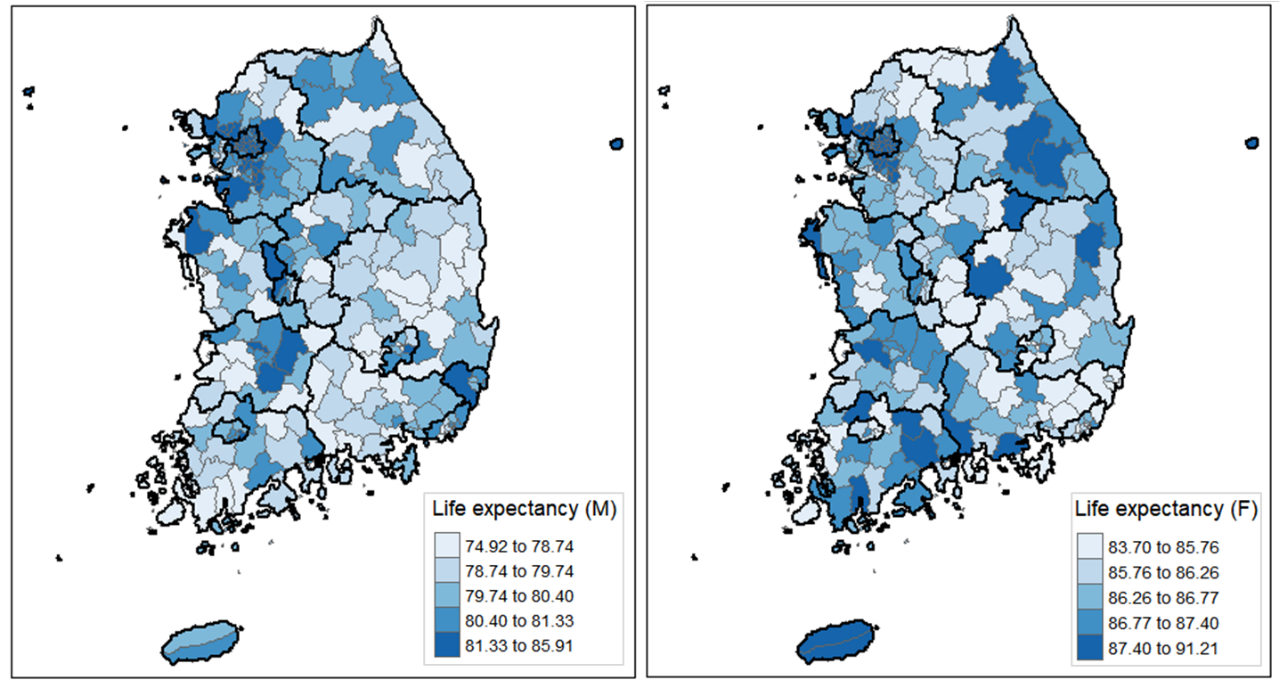


## Figure A4. Life expectancy at age of 65 by quintiles of deprivation index during 2012~2021 (by sex)


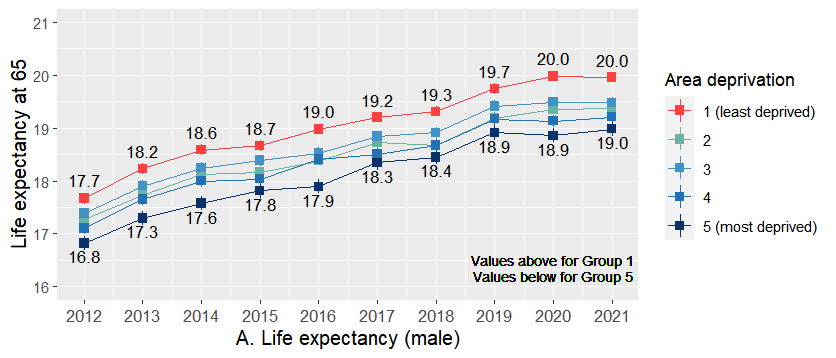


**
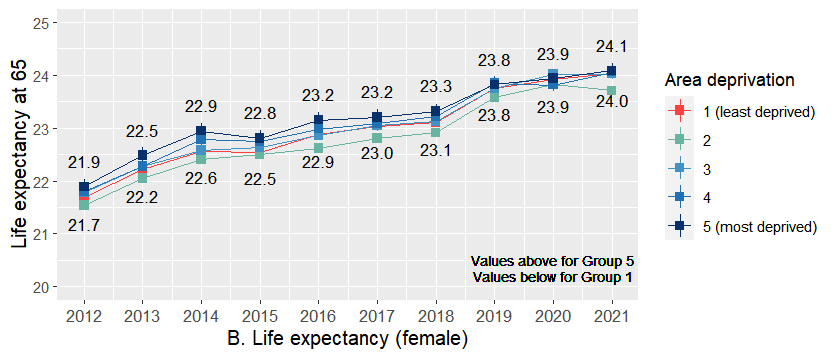
**

**Figure A5. The trend of slope of inequality index for life expectancy at birth by quintiles of deprivation index, 2012 ~ 2012**

**
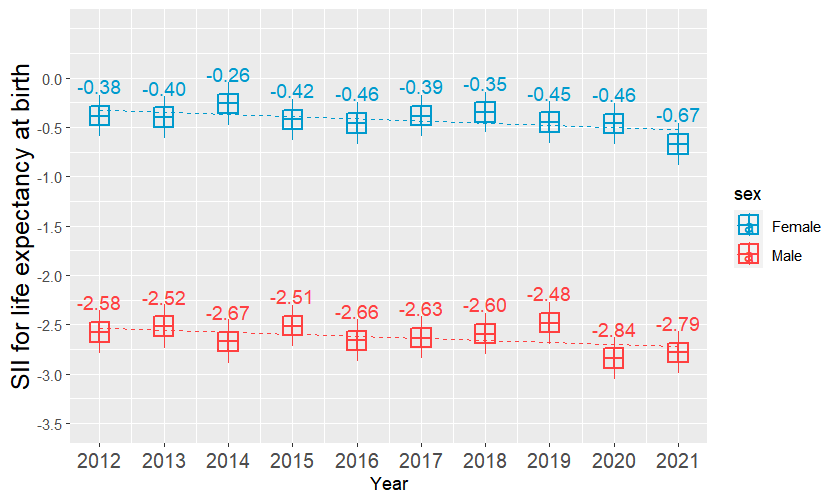
**

## Figure A6. Number of years contributed by age and cause of deaths to the life expectancy gap betweeen the most deprived and the least deprived areas, 2015 ~ 2019 and 2020 ~ 2021 (Male)


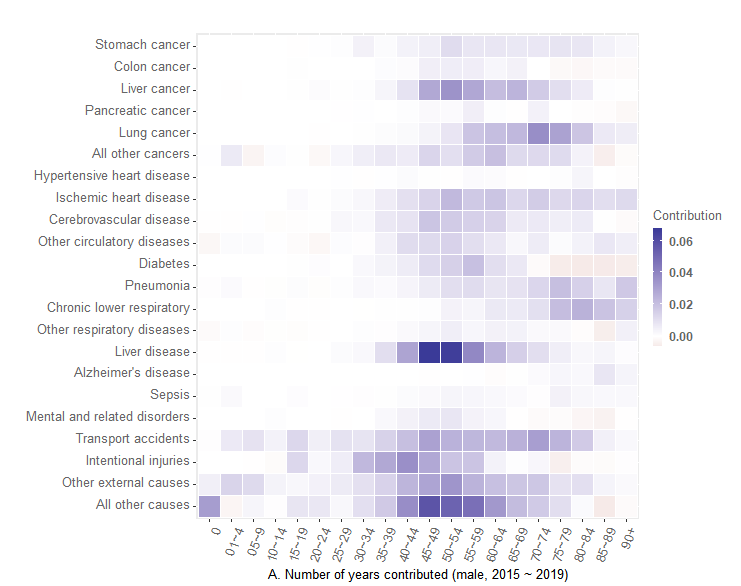


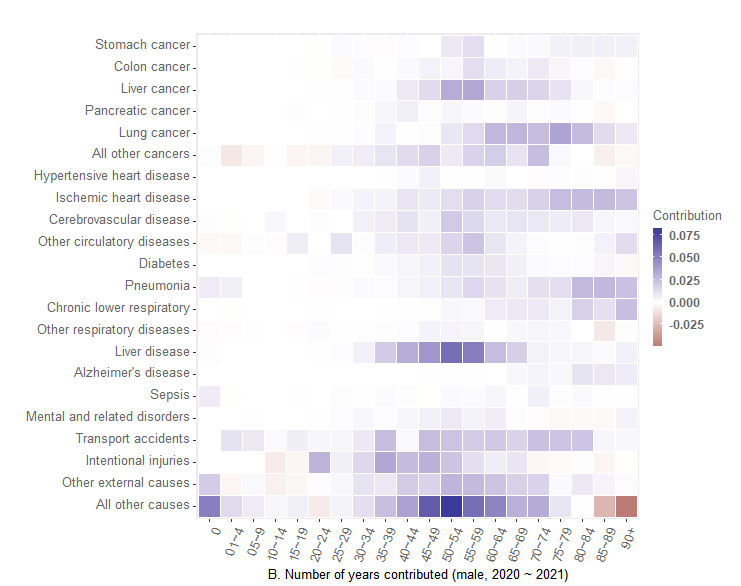


Note: A positive value indicates a contribution from the most deprived area (i.e., greater mortality).

## Figure A7. Number of years contributed by age and cause of deaths to the life expectancy gap betweeen the most deprived and the least deprived areas, 2015 ~ 2019 and 2020 ~ 2021 (Female)


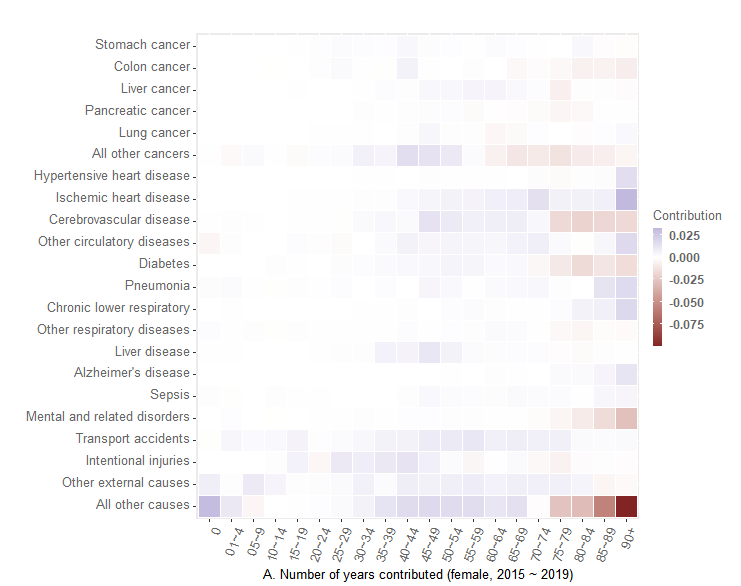


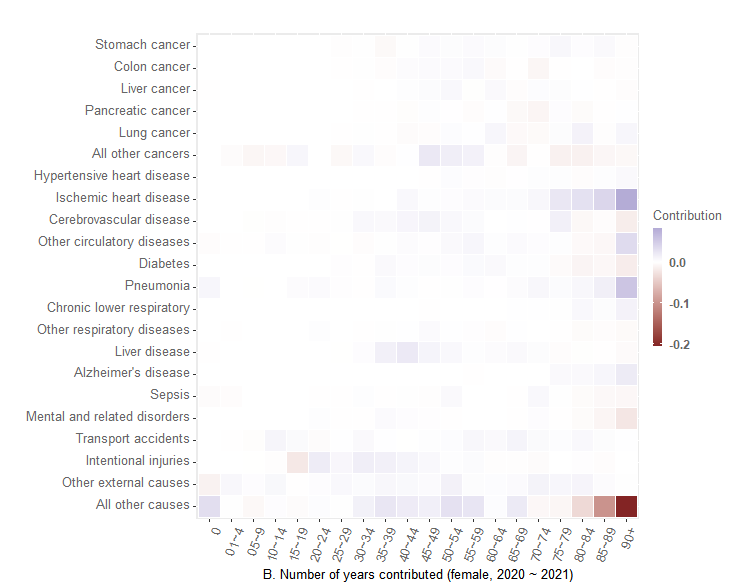


Note: A positive value indicates a contribution from the most deprived area (i.e., greater mortality).
